# Urinary Paraben Concentrations and Ovarian Aging among Women from a Fertility Center

**DOI:** 10.1289/ehp.1205350

**Published:** 2013-08-02

**Authors:** Kristen W. Smith, Irene Souter, Irene Dimitriadis, Shelley Ehrlich, Paige L. Williams, Antonia M. Calafat, Russ Hauser

**Affiliations:** 1 Department of Environmental Health, Harvard School of Public Health, Boston, Massachusetts, USA; 2 Department of Obstetrics and Gynecology, Division of Reproductive Endocrinology and Infertility, Harvard Medical School/Massachusetts General Hospital Fertility Center, Boston, Massachusetts, USA; 3 Department of Biostatistics, Harvard School of Public Health, Boston, Massachusetts, USA; 4 National Center for Environmental Health, Centers for Disease Control and Prevention, Atlanta, Georgia, USA

## Abstract

Background: Parabens are preservatives commonly used in personal care products, pharmaceuticals, and foods. There is documented widespread human exposure to parabens, and some experimental data suggest that they act as estrogenic endocrine disruptors. As far as we are aware, no epidemiologic studies have assessed female reproductive health effects in relation to paraben exposure.

Objective: We examined the association of urinary paraben concentrations with markers of ovarian reserve in a prospective cohort study of women seeking fertility treatment at Massachusetts General Hospital, Boston, Massachusetts.

Methods: Measures of ovarian reserve were day-3 follicle-stimulating hormone (FSH), antral follicle count (AFC), and ovarian volume. Paraben concentrations [methylparaben (MP), propylparaben (PP), and butylparaben (BP)] were measured in spot urine samples collected prior to the assessment of outcome measures. We used linear and Poisson regression models to estimate associations of urinary paraben concentrations (in tertiles) with ovarian reserve measures.

Results: Of the women enrolled in 2004–2010, 192 had at least one ovarian reserve outcome measured (mean age ± SD, 36.1 ± 4.5 years; range, 21.0–46.7 years). MP and PP were detected in > 99% of urine samples and BP in > 75%. We found a suggestive trend of lower AFC with increasing urinary PP tertiles [mean percent change (95% CI) for tertiles 2 and 3 compared with tertile 1, respectively, were –5.0% (–23.7, 18.4) and –16.3% (–30.8, 1.3); trend *p*-value (*p*_trend_) = 0.07] as well as higher day-3 FSH with higher urinary PP tertiles [mean change (95% CI) for tertiles 2 and 3 compared with tertile 1 were 1.16 IU/L (–0.26, 2.57) and 1.02 IU/L (–0.40, 2.43); *p*_trend_ = 0.16]. We found no consistent evidence of associations between urinary MP or BP and day-3 FSH or AFC, or between urinary MP, PP, or BP and ovarian volume.

Conclusions: PP may be associated with diminished ovarian reserve. However, our results require confirmation in further studies.

Citation: Smith KW, Souter I, Dimitriadis I, Ehrlich S, Williams PL, Calafat AM, Hauser R. 2013. Urinary paraben concentrations and ovarian aging among women from a fertility center. Environ Health Perspect 121:1299–1305; http://dx.doi.org/10.1289/ehp.1205350

## Introduction

Parabens are a family of chemicals commonly used as antimicrobial preservatives in personal care products, pharmaceuticals, and foods (Andersen 2008; National Toxicology Program 2005; Orth 1980). Exposure to parabens can occur through ingestion, inhalation, or dermal absorption. Following excretion, the parent compounds can be measured in urine and have been shown to be valid biomarkers of exposure (Ye et al. 2006a).

Although parabens are quickly eliminated from the body (Janjua et al. 2008), they have been detected in the general U.S. population (Calafat et al. 2010; Ye et al. 2006a). Methylparaben (MP) and propylparaben (PP), the two most commonly used parabens (Soni et al. 2005), were detected in > 92% of a representative sample of the U.S. population in the National Health and Nutrition Examination Survey (NHANES), whereas butylparaben (BP) was detected in 47% of participants (Calafat et al. 2010). Parabens have been detected in urine samples collected from infants (Calafat et al. 2009) and older children (Calafat et al. 2010; Casas et al. 2011; Wolff et al. 2010), in adults of reproductive age and older (Calafat et al. 2010; Meeker et al. 2011), and in pregnant women (Casas et al. 2011; Philippat et al. 2012; Smith et al. 2012), suggesting that exposure to parabens is ubiquitous and may begin in early life and extend throughout the lifespan.

Parabens are suspected endocrine disruptors; they are estrogenic (Golden et al. 2005; Routledge et al. 1998; Soni et al. 2005), although they have a lower estrogen receptor binding affinity than does endogenous estrogen (Routledge et al. 1998; Vo et al. 2010). Parabens have been shown to bind to both estrogen receptor (ER)-α and ER-β (Gomez et al. 2005; Okubo et al. 2001). The estrogenic activity of parabens increases with increasing length and branching of the alkyl chain (e.g., BP > PP > MP) (Byford et al. 2002; Routledge et al. 1998; Vo et al. 2010).

On the basis of available toxicologic data, MP and PP were classified as generally regarded as safe (GRAS) in 1972 by the U.S. Food and Drug Administration (FDA 2013). In 2008, the Cosmetic Ingredient Review Panel concluded that parabens used in cosmetics, including BP, do not pose a safety risk based on the available data (Andersen 2008).

A few recent animal toxicity studies have reported adverse effects of some parabens on female reproductive and endocrine function (Kang et al. 2002; Taxvig et al. 2008; Vo et al. 2010). In one study evaluating pre-pubertal female rats treated orally with parabens, effects included—but were not limited to—a decrease in ovarian weight and histopathological changes in the ovaries, as well as altered estradiol and tetraiodothyronine (T_4_), but not thyroid-stimulating hormone (TSH) levels (Vo et al. 2010). In that study, effects were seen with MP, BP, isopropylparaben, and isobutylparaben, and the relationships varied by outcome, some of which were dose dependent.

In a study evaluating pregnant rats exposed subcutaneously to parabens, together with their prenatally exposed fetuses, Taxvig et al. (2008) observed a decrease in ER-β expression in the ovaries of BP-exposed female fetuses. (*ER-*β gene expression was significantly decreased in animals exposed to either of the BP doses administered compared with the control, but it is unclear whether gene expression differed between the BP doses.) However, these researchers observed no change in ovarian estradiol levels or ovarian histopathology. In addition, Taxvig et al. (2008) found no association of maternal or fetal reproductive hormone levels with ethylparaben or BP exposure. In another study of pregnant rats exposed subcutaneously to BP, Kang et al. (2002) found no evidence of effects on reproductive organ weights and no histopathological abnormalities in female offspring.

Overall, these limited studies suggest that some parabens may exert adverse endocrine-disrupting effects on female animals, but additional toxicologic data, including mechanistic studies, are needed. Human data on the reproductive health effects of paraben exposure are limited, and as far as we are aware, no studies have reported on the association of urinary paraben concentrations with female reproductive health outcomes.

Given the suspected endocrine-disrupting properties of parabens and the sensitivity of oogenesis to proper estrogen signaling, we were interested in evaluating the potential association between urinary paraben concentrations and markers of ovarian reserve. Hormonal and ultrasonographic markers of ovarian reserve are commonly used by reproductive endocrinology and infertility specialists to evaluate a woman’s response to ovarian stimulation, and include serum concentration of follicle-stimulating hormone (FSH) on day 3 of the menstrual cycle, antral follicle count (AFC), and ovarian volume (OV). Typically, as a woman’s age increases, her ovarian reserve diminishes (“ovarian aging”); this is associated with reduced fertility. Among women undergoing assisted reproductive technology (ART), ovarian aging is also associated with a decreased response to ovarian stimulation protocols and lower pregnancy success rates (Elter et al. 2005; Levi et al. 2001). This diminished ovarian reserve is generally indicated by higher day-3 FSH levels and lower AFC and OV. However, there are factors other than age that could be associated with a diminished ovarian reserve, possibly including exposure to endocrine-disrupting chemicals. Therefore, the objective of this study was to evaluate whether exposure to parabens, assessed from urinary paraben concentrations, is associated with diminished ovarian reserve among women undergoing *in vitro* fertilization or intrauterine insemination.

## Methods

*Participants.* Study participants were female patients from the Massachusetts General Hospital (MGH) Fertility Center who were undergoing infertility evaluation and participating in our ongoing prospective cohort study on environmental risk factors for reproductive health (Environment and Reproductive Health Study). The participants had at least one hormonal or ultrasonographic marker of ovarian reserve measured (day-3 FSH, AFC, or OV) and also contributed at least one urine sample for the measurement of paraben concentrations prior to the measurement of the markers of ovarian reserve. All female patients > 18 years of age and < 46 years (at enrollment) seeking infertility evaluation or treatment at the MGH Fertility Center were eligible to participate (close to 100% of patients were eligible) and approximately 60% consented. We excluded participants who previously had an oophorectomy (*n* = 5). We recruited participants between December 2004 and October 2010 and followed them from study entry until the discontinuation of fertility treatment, a live birth, or loss to follow-up. Two patients reenrolled in the study after the end of the initial follow-up period; only data from their first enrollment were included in this analysis. The study was approved by the Human Studies Institutional Review Boards of the MGH, Harvard School of Public Health (HSPH), and the Centers for Disease Control and Prevention (CDC). Participants signed an informed consent after the study procedures were explained by a research nurse and all questions were answered.

*Clinical data.* Clinical information was abstracted from the patient’s electronic medical record by a research nurse. An intravenous blood sample was drawn on the third day of the menstrual cycle, and the serum was analyzed for FSH with an automated electrochemiluminescence immunoassay at the MGH Core Laboratory, as previously described (Mok-Lin et al. 2010). AFC and OV were measured for both ovaries by a reproductive endocrinology and infertility specialist at the MGH Fertility Center using transvaginal ultrasound. We calculated the OV using the following formula: [length (millimeters) × width (millimeters) × height (millimeters)] × (π/6). We used the sum of antral follicles from the left and right ovaries (AFC) and the average volume of left and right ovaries (OV) in the analysis. Subsequent to an infertility evaluation, each patient was given an infertility diagnosis by a physician at the MGH Fertility Center according to the Society for Assisted Reproductive Technology (SART) definitions, as previously described (Mok-Lin et al. 2010). The participant’s date of birth and demographic characteristics were collected using a nurse-administered questionnaire at entry into the study, and weight and height were measured by the nurse.

*Urinary paraben measurements.* We collected a convenience spot urine sample from the women at the time of recruitment and at subsequent visits during infertility treatment cycles. Although participants were recruited into this study beginning in 2004, the measurement of parabens in urine did not begin until August 2005, when these chemicals were added to the study protocol. We collected samples between August 2005 and November 2010. Urine was collected in a sterile polypropylene cup. After measuring specific gravity (SG) using a handheld refractometer (National Instrument Company, Inc., Baltimore, MD), the urine was divided into aliquots and frozen at –80°C. Samples were shipped on dry ice overnight to the CDC, where concentrations of total (free plus conjugated) MP, PP, and BP were measured using online solid-phase extraction–high performance liquid chromatography–isotope dilution tandem mass spectrometry, as previously reported (Ye et al. 2006b). Standard QA/QC procedures were followed (CDC 2010b). The limits of detection (LODs) were 1.0 μg/L for MP and 0.2 μg/L for PP and BP.

*Statistical analysis.* Demographic characteristics of the study participants (mean and percentage) are reported separately for each outcome measure because the number of participants varied by outcome. These characteristics are also reported for participants with any ovarian reserve outcome measured. The distribution of day-3 FSH, AFC, and OV are described using the mean ± SD, median and interquartile range (IQR), and range. We computed the within-person geometric mean (GM) of all urinary paraben concentrations (MP, PP, and BP) measured prior to outcomes as a summary exposure measure for each participant. We summarized the distribution of exposures using the median, IQR, and range of urinary paraben concentrations. We assigned urinary concentrations below the LOD with a value equal to the LOD divided by the square root of two (Hornung and Reed 1990). We corrected urinary paraben concentrations for SG using a modification of a previously described formula (Duty et al. 2005):

*Pc* = *P*[(1.016 – 1)/SG – 1],

where *Pc* is the SG-corrected paraben concentration (micrograms per liter), *P* is the measured paraben concentration (micrograms per liter), and 1.016 is the mean (and median) SG level in the study population. We used SG-corrected paraben concentrations in all analyses. We calculated the Spearman correlation (*r*_S_) between the within-person GMs of the different parabens.

We calculated the *r*_S_ between the markers of ovarian reserve (day-3 FSH, AFC, and OV), age, and body mass index (BMI; kilograms per meter squared). Among women with outcome measures available for each pair of correlations, we calculated the correlation between FSH and AFC, FSH and OV, and AFC and OV. We calculated the correlation of age and BMI with each separate outcome measure using all available measurements. We were interested in evaluating the association between BMI and these measures of ovarian reserve because a higher BMI has been shown to be associated with infertility (Pasquali et al. 2003; Shah et al. 2011), although there is limited evidence in the literature for an association between ovarian reserve measures and BMI (Su et al. 2008).

We used multivariable linear regression to estimate associations between within-person MP, PP, and BP GM concentrations (divided into tertiles) with day-3 FSH and OV. OV was natural log (ln)-transformed [ln(OV)] prior to all regression analyses to reduce skewness. We used Poisson regression to estimate associations between within-person MP, PP, and BP GM concentrations (divided into tertiles) with AFC. Covariates considered for inclusion in the regression models included age in years at the time of the outcome measure and BMI at entry into the study (both modeled as continuous measures); these were also included when related to the outcome measure in univariate regression models (*p* < 0.20). We considered age categorized into < 37 years and ≥ 37 years for inclusion as a covariate in a sensitivity analysis because the ability to become pregnant declines around 37 years of age among women in the U.S. population undergoing ART (CDC 2010a). To allow for easier interpretation of the results, we exponentiated the parameter estimates for the linear regression model evaluating ln(OV) and for the Poisson regression model evaluating ln(AFC). The mean percent change in the outcome from the lowest tertile of paraben concentrations is presented for these two outcomes (OV and AFC). We conducted all tests for trend by assigning each urinary paraben concentration tertile an ordinal integer value of 0 (lowest tertile) to 2 (highest tertile).

As a sensitivity analysis, we reran the regression models for AFC and OV excluding patients diagnosed with polycystic ovarian syndrome (PCOS) because these women tend to have a higher AFC and larger OV than women without this disease. PCOS was defined using a SART diagnosis (primary, secondary, or tertiary) of ovulatory disorder.

In a secondary analysis, we combined the parabens using two methods. We first used an estrogen equivalency (EEQ) factor approach (Safe 1998; Shirai et al. 2012) using the following formula:

EEQ(parabens) = (*MP*_m_ × 1) + (*PP*_m_ × 83.3) + (*BP*_m_ × 250),

where *MP*_m_, *PP*_m_, and *BP*_m_ are the SG-adjusted within-person GM molar concentrations, and the potency factors come from an *in vitro* yeast-based estrogen assay (BP, ~ 10,000 times less potent than 17β-estradiol; PP, ~ 30,000 times less potent; and MP ~ 2,500,000 less potent) (Routledge et al. 1998). Second, we summed the urinary paraben concentrations using the following formula:

Σ(parabens) = *MP*_m_ + *PP*_m_ + *BP*_m_.

We used multivariable linear regression to separately evaluate the association between EEQ(parabens) and Σ(parabens) (both divided into tertiles) with day-3 FSH and OV. OV was ln-transformed prior to all regression analyses to reduce skewness. We used Poisson regression to analyze the association between EEQ(parabens) and Σ(parabens) (both divided into tertiles) with AFC. We conducted all statistical analyses using SAS, version 9.2 (SAS Institute Inc., Cary, NC) and considered two-sided significance levels < 0.05 statistically significant.

This exploratory study provided 80% power to detect a difference of 2.11 IU/L in day-3 FSH or 0.38 mm^3^ in ln(OV), for comparing high or medium urinary paraben concentrations with low urinary paraben concentrations (0.67 SDs). Similarly, the study design provided 80% power for detecting a difference in AFC of 4.2, corresponding to a decrease of approximately 38% between high and low paraben urinary concentrations.

## Results

A total of 193 women had at least one measure of ovarian reserve available and at least one urinary paraben concentration measurement. We excluded one woman for which all urine samples had missing SG measurements, resulting in a final sample of 192 women. Because all outcome measures were not available for all participants, we evaluated each outcome separately using all available measurements: day-3 FSH was measured in 110 women, AFC in 142 women, and OV in 109 women. There were 44 women with all three measures, 81 women with two measures, and 67 women with only one measure available. We collected 1–14 urine samples from each participant (median in each data set, 1 sample/participant; range of means in all three data sets, 2.2–2.6 samples/participant) that contributed to the GM summary exposure measure. The urine samples were collected between 0 (the same day) and 1,145 days before the outcome measure for the AFC data set (mean ± SD, 142 ± 182 days; median, 94 days), and between 0 and 981 days before the FSH (mean ± SD, 157 ± 159 days; median, 108 days) and OV (mean ± SD, 110 ± 128 days; median, 77 days) data sets.

Women were primarily Caucasian, non-smokers, and > 35 years of age, and had a mean BMI (± SD) of 25.4 ± 5.15 (Table 1). The SART diagnosis was most commonly female factor, followed by male factor and unexplained infertility (Table 1). There was no significant difference in age or the number of participants diagnosed with PCOS in each of the three outcome subgroups (data not shown). The mean day-3 FSH was 7.39 ± 3.17 IU/L with a range of 0.10–26.0 IU/L. The median (IQR) AFC sum (left and right ovaries) was 11 (7–15) with a range of 2–40. The median (IQR) OV was 4,928 mm^3^ (3,634–7,588) with a range of 1,359–27,834 mm^3^.

**Table 1:** Characteristics by ovarian reserve outcome of 192 women participants of a prospective fertility study at Massachusetts General Hospital enrolled between 2004 and 2010.

Characteristic	Day-3 FSH (*n* = 110)	AFC (*n*= 142)	OV (*n* = 109)	Any ovarian reserve outcome measured (*n* = 192)
Age (years)
Mean ± SD	36.1 ± 4.67	36.3 ± 4.24	35.6 ± 4.64	36.1 ± 4.48^*a*^
Range	22.0–45.3	21.0–44.8	21.7–46.7	21.0–46.7
BMI (kg⁄m^2^)^*b*^
Mean ± SD	25.6 ± 5.54	25.3 ± 5.14	24.9 ± 4.70	25.4 ± 5.15
Range	17.3–42.4	17.5–40.5	17.5–40.5	17.3–42.4
Race [*n *(%)]
Caucasian	85 (77)	115 (81)	92 (84)	156 (81)
African American/black	8 (7)	6 (4)	5 (5)	10 (5)
Asian	5 (5)	9 (6)	7 (6)	10 (5)
Native American/Alaska Native	1 (1)	1 (1)	0 (0)	1 (1)
Other	11 (10)	11 (8)	5 (5)	15 (8)
Smoking history [*n *(%)]
Never	86 (78)	106 (75)	76 (70)	142 (74)
Former	20 (18)	29 (20)	27 (25)	42 (22)
Current	4 (4)	7 (5)	6 (5)	8 (4)
SART diagnosis [*n *(%)]^*c*^
Female factor	48 (44)	60 (43)	40 (37)	81 (42)
Endometriosis	8 (7)	8 (6)	8 (7)	12 (6)
Tubal factor	8 (7)	10 (7)	5 (5)	13 (7)
Diminished ovarian reserve	14 (13)	18 (13)	9 (8)	23 (12)
Ovulation disorders	17 (16)	23 (16)	18 (17)	31 (16)
Uterine disorders	1 (1)	1 (1)	0 (0)	2 (1)
Male factor	31 (28)	32 (23)	30 (28)	49 (26)
Unexplained	23 (21)	40 (28)	31 (29)	50 (26)
Other	7 (6)	9 (6)	7 (6)	11 (6)
^***a***^For women with more than one outcome measure, the woman’s age at each outcome measure was averaged, and this value was used to calculate the mean ± SD. ^***b***^FSH data set, *n* = 109; OV data set, *n* = 108; among all women with any outcome measured, *n* = 191. ^***c***^Primary SART diagnosis; FSH data set, *n* = 109; AFC data set, *n* = 141; OV data set, *n* = 108; among all women with any outcome measured, *n* = 191.				

Urinary paraben concentrations were similar to those in the general population; MP and PP were detected in > 99% of samples, and BP in > 75% (Table 2). In each of the three data sets, there was a strong correlation between concentrations of MP and PP (*r*_S_ range, 0.81–0.85) and a moderate correlation for MP and BP (*r*_S_ range, 0.40–0.47) and PP and BP (*r*_S_ range, 0.43–0.46).

**Table 2:** Distribution of urinary paraben concentrations (μg/L) measured among participants of a prospective fertility study at Massachusetts General Hospital enrolled between 2004 and 2010, by ovarian reserve outcome.

Paraben	*n*	Percent detected^*a*^	Uncorrected concentration			SG-corrected concentration		
			Minimum	Median (IQR)	Maximum	Minimum	Median (IQR)	Maximum
Day 3 FSH
MP	110	100.0	6.70	210 (75.2, 520)	4,400	10.8	249 (89.0, 549)	2,428
PP	110	99.7	0.20	49.6 (13.0, 89.3)	1,000	0.46	55.1 (22.5, 124)	727
BP	110	80.0	< LOD	2.08 (0.40, 6.58)	142	< LOD	2.83 (0.40, 9.60)	177
AFC
MP	142	100	3.00	180 (74.7, 400)	4,400	5.13	227 (84.4, 492)	2,428
PP	142	99.4	< LOD	37.1 (17.4, 83.4)	1,430	< LOD	52.1 (21.5, 110)	727
BP	142	78.4	< LOD	1.42 (0.30, 6.07)	142	< LOD	1.60 (0.33, 9.49)	177
OV
MP	109	100.0	3.00	158 (57.7, 343)	4,400	7.77	219 (72.2, 429)	2,428
PP	109	99.2	< LOD	35.5 (11.6, 88.7)	1,430	< LOD	61.0 (14.6, 110)	654
BP	109	75.8	< LOD	1.53 (0.30, 6.13)	142	< LOD	2.18 (0.45, 8.50)	102
LODs were 1.0 μg/L for MP and 0.2 μg/L for PP and BP. ^***a***^Percentage of concentrations > LOD. Total samples with PP concentrations < LOD by data set: 1 of 290 (FSH), 2 of 310 (AFC), 2 of 252 (OV); total samples with BP concentrations < LOD by data set: 58 of 290 (FSH), 67 of 310 (AFC), 61 of 252 (OV).								

We assessed the correlation of day-3 FSH, AFC, and OV among women having outcome measures for each pair of correlations. Among women with both FSH and AFC (*n* = 85), FSH was negatively correlated with AFC (*r* = –0.40, *p* = 0.002); among women with both FSH and OV (*n* = 49), FSH was negatively correlated with OV (*r* = –0.36, *p* = 0.01); and among women with both AFC and OV (*n* = 79), AFC was positively correlated with OV (*r* = 0.47, *p* < 0.0001). FSH was positively correlated with age (*r* = 0.29, *p* = 0.002) but not with BMI (*r* = –0.048, *p* = 0.62). AFC and OV were negatively correlated with age (*r* = –0.44, *p* < 0.001; and *r* = –0.21, *p* = 0.025, respectively) but not with BMI (*r* = 0.036, *p* = 0.67; and *r* = 0.040, *p* = 0.68, respectively).

PP concentration was positively related to day-3 FSH, with mean day-3 FSH higher in tertiles 2 and 3 than in tertile 1, although there was not a significantly increasing trend across tertiles: The mean level in tertile 3 was similar to that in tertile 2 [trend *p*-value (*p*_trend_) = 0.16]. For MP and BP, mean day-3 FSH was also higher in tertiles 2 and 3 than in tertile 1, although the mean levels of both analytes were lower in tertile 3 compared with tertile 2 (*p*_trend_ of 0.64 and 0.60 for MP and BP, respectively) (Table 3). We observed a suggestive trend for lower AFC among women with higher PP concentrations, with the mean percent difference from tertile 1 in AFC decreasing across tertiles (*p*_trend_ = 0.07). For MP, the magnitude of the parameter estimates in tertiles 2 and 3 was similar to PP, although the trend was not significant (*p*_trend_ = 0.31). We observed no association between BP and AFC (Table 4). There was no evidence of an association between urinary MP, PP, or BP concentration and OV (Table 5). Age was significantly negatively associated with AFC and OV and positively associated with FSH; thus, age was included as a covariate in all regression models as a continuous measure (age was not associated with the exposures), whereas BMI was not observed to be associated (*p* > 0.20) and was not included.

**Table 3:** Estimated mean change in day‑3 FSH (IU/L) by urinary paraben concentration tertile from linear regression models.

Paraben concentration (μg/L)	*n*	Estimated mean change in FSH (95% CI)^*a*^	*p*-Value
MP
Tertile 3 (432–2,428)	37	0.35 (–1.07, 1.77)	0.63
Tertile 2 (154–430)	37	1.04 (–0.39, 2.46)	0.15
Tertile 1 (10.8–144)	36	0 (Reference)
*p*_trend _		0.64
PP
Tertile 3 (87.8–727)	37	1.02 (–0.40, 2.43)	0.16
Tertile 2 (32.0–80.9)	37	1.16 (–0.26, 2.57)	0.11
Tertile 1 (0.46–29.4)	36	0 (Reference)
*p*_trend _		0.16
BP
Tertile 3 (6.00–177)	37	0.39 (–1.03, 1.82)	0.59
Tertile 2 (0.96–5.61)	37	0.95 (–0.48, 2.39)	0.19
Tertile 1 (< LOD–0.85)	36	0 (Reference)
*p*_trend _		0.60
All model results were adjusted for age; paraben concentrations were SG-corrected. LODs were 1.0 μg/L for MP and 0.2 μg/L for PP and BP. ^***a***^Parameter estimates can be interpreted as an IU/L change in day-3 FSH for each tertile of urinary paraben concentration relative to tertile 1 (reference). For example, in tertile 3 of the PP urinary concentrations, there is a 1.02-IU/L increase, on average, in day-3 FSH compared with tertile 1.			

**Table 4:** Estimated mean percent change in AFC by urinary paraben concentration tertile based on the Poisson regression model.

Paraben concentration (μg/L)	*n*	Estimated mean percent change in AFC (95% CI)	*p*-Value
MP
Tertile 3 (381–2,428)	47	–10.6 (–28.2, 11.2)	0.31
Tertile 2 (145–377)	48	–6.8 (–23.5, 13.7)	0.49
Tertile 1 (5.13–132)	47	0 (Reference)
*p*_trend _		0.31
PP
Tertile 3 (87.8–727)	47	–16.3 (–30.8, 1.3)	0.07
Tertile 2 (26.3–81.8)	48	–5.0 (–23.7, 18.4)	0.65
Tertile 1 (< LOD–25.2)	47	0 (Reference)
*p*_trend _		0.07
BP
Tertile 3 (5.44–177)	48	–2.0 (–21.0, 21.6)	0.86
Tertile 2 (0.75–5.12)	47	–4.8 (–22.5, 16.8)	0.63
Tertile 1 (< LOD–0.73)	47	0 (Reference)
*p*_trend _		0.86
All model results were adjusted for age; paraben concentrations were SG-corrected. LODs were 1.0 μg/L for MP and 0.2 μg/L for PP and BP.			

**Table 5:** Estimated mean percent change in OV by urinary paraben concentration tertile based on the linear regression model.

Paraben concentration (μg/L)	*n*	Estimated mean percent change in OV (95% CI)	*p*-Value
MP
Tertile 3 (332–2,428)	36	4.7 (–19.4, 35.9)	0.73
Tertile 2 (100–326)	37	15.0 (–11.2, 49.1)	0.29
Tertile 1 (7.77–95.7)	36	0 (Reference)
*p*_trend _		0.73
PP
Tertile 3 (87.8–654)	36	–0.9 (–23.5, 28.3)	0.94
Tertile 2 (27.1–81.8)	37	19.2 (–8.0, 54.4)	0.18
Tertile 1 (< LOD–25.2)	36	0 (Reference)
*p*_trend _		0.94
BP
Tertile 3 (6.02–102)	36	–4.2 (–26.1, 24.2)	0.74
Tertile 2 (0.82–5.43)	36	1.0 (–22.2, 31.2)	0.94
Tertile 1 (< LOD–0.80)	37	0 (Reference)
*p*_trend _		0.74
All model results were adjusted for age; paraben concentrations were SG-corrected. LODs were 1.0 μg/L for MP and 0.2 μg/L for PP and BP.			

In a sensitivity analysis controlling for age categorized into < 37 years and ≥ 37 years, the association between day-3 FSH and PP became stronger: The mean difference in day-3 FSH was 1.24 IU/L [95% confidence interval (CI): –0.18, 2.67] in tertile 3 [using age as a continuous covariate, the mean difference was 1.02 (95% CI: –0.40, 2.43)], and 1.19 IU/L (95% CI: –0.22, 2.60) in tertile 2 [using age as a continuous covariate, the mean difference was 1.16 (95% CI: –0.26, 2.57)], both compared with tertile 1 (with the *p*_trend_ of 0.08 becoming borderline significant). Including the categorized age variable, the association between day-3 FSH and MP became stronger, with a mean difference in day-3 FSH of 0.57 IU/L (95% CI: –0.85, 1.99) in tertile 3, and of 1.21 IU/L (95% CI: –0.21, 2.63) in tertile 2, both compared with the lowest tertile. Including the categorized age variable, the association between day-3 FSH and BP became stronger with a mean difference in day-3 FSH of 0.53 IU/L (95% CI: –0.89, 1.95) in tertile 3, and of 1.10 IU/L (95% CI: –0.32, 2.52) in tertile 2, both compared with tertile 1. The *p*_trend_ values for MP and BP remained nonsignificant when the categorized age variable was included. The relationship of MP, PP, and BP with AFC and OV was similar when including the categorized age variable compared with the continuous age variable (data not shown).

Among patients with an available SART diagnosis (*n* = 1 missing from each data set), excluding PCOS patients (*n* = 25 in the AFC data set; *n* = 20 in the OV data set) and controlling for age (continuous), the relationship of PP with AFC was attenuated (data not shown). Excluding PCOS patients altered parameter estimates for associations of MP and BP with AFC and associations of MP, PP, and BP with OV, but estimates were imprecise and remained nonsignificant (data not shown).

In a secondary exploratory analysis evaluating the association of combined concentrations of parabens with the three ovarian reserve outcomes (controlling for age as a continuous measure), we found that EEQ(parabens) was negatively related to AFC in tertile 3, with a mean difference of –6.4% (95% CI: –24.4, 15.8), and little evidence of an association in tertile 2, with a mean difference of 1.1% (95% CI: –17.3, 23.5), both compared with tertile 1, although the trend was not statistically significant (*p*_trend_ = 0.54) (see Supplemental Material, Table S1). Similarly, we found that Σ(parabens) was negatively related to AFC, with a mean difference of –10.8% (95% CI: –28.2, 10.7) in tertile 3, and of –6.5% (95% CI: –23.4, 14.1) in tertile 2, both compared with tertile 1, although the trend was not statistically significant (*p*_trend_ = 0.30). We found no significant relationships between EEQ(parabens) or Σ(parabens) with day-3 FSH or OV (see Supplemental Material, Tables S2 and S3, respectively).

## Discussion

As far as we are aware, this is the first epidemiologic study to assess female reproductive health outcomes in relation to biomarkers of paraben exposure. We found that nearly 100% of the women included in this study had detectable urinary concentrations of MP and PP, and > 75% of women had detectable BP concentrations. Urinary paraben concentrations were similar to those reported for all females from NHANES in 2005–2006 (Calafat et al. 2010). In that study, median (IQR) unadjusted urinary paraben concentrations for women were 137 μg/L (35.4–356 μg/L), 29.1 μg/L (5.30–93.0 μg/L), and 0.50 μg/L (< LOD–3.70 μg/L) for MP, PP, and BP, respectively (Calafat et al. 2010). We found suggestive evidence of a negative relationship between urinary PP and AFC, considered one of the best markers of ovarian reserve (Rosen et al. 2012). Higher urinary PP was associated with a higher day-3 FSH, which is consistent with PP’s negative association with AFC. The positive relationship of urinary PP with day-3 FSH approached statistical significance when controlling for age categorized into < 37 years and ≥ 37 years, although the magnitude of the association was similar as when controlling for age as a continuous measure. These findings suggest that exposure to PP may adversely affect ovarian reserve, and thus contribute to ovarian aging, among women attending a fertility clinic. Although evidence of a negative relationship between MP and AFC was suggestive, there was no clear evidence of associations between urinary MP or BP concentrations with any of the markers of ovarian reserve. Similar to the relationship between urinary PP and AFC, in an exploratory analysis the three parabens combined were negatively related to AFC using both EEQ(parabens) and Σ(parabens), although the relationship did not approach statistical significance.

It has been established that parabens are estrogenic (Golden et al. 2005; Routledge et al. 1998; Soni et al. 2005) and that they bind to both ER-α and ER-β (Gomez et al. 2005; Okubo et al. 2001). Although the estrogen receptor binding affinity of parabens is much lower than that of endogenous estrogen (Darbre and Harvey 2008), oogenesis is highly dependent on proper estrogen signaling (Hewitt et al. 2005); therefore, even slight changes in the ovarian hormonal environment (either *in utero* or later in life) could contribute to altered ovarian function. The relationship of PP with diminished ovarian reserve is consistent with animal data showing that the estrogenicity of parabens, and therefore the potential for reproductive toxicity, is greater in PP compared with MP (Byford et al. 2002; Routledge et al. 1998; Vo et al. 2010). Although the animal data also show that BP is more estrogenic than PP or MP, we detected BP less frequently; when it was detected, urinary concentrations of BP were much lower than those of either PP or MP, which may explain the lack of an association of BP with markers of ovarian reserve. It is also possible that biological activity and mechanisms of action differ between the parabens. However, as far as we know, this has not been studied.

A few studies conducted in female rats and mice have suggested an association between paraben exposure and reproductive outcomes (Taxvig et al. 2008; Vo et al. 2010). These outcomes include changes in ovarian weight and histopathology, as well as changes in *ER*-α and *ER*-β gene expression. In a study of prepubertal female mice treated orally with parabens, adverse effects included a decrease in ovarian weight (MP and isopropyl-paraben, but not PP or BP) and histopathological changes in the ovaries (MP, isopropyl-paraben, BP, and isobutyl-paraben, but not PP) (Vo et al. 2010). These histopathological changes included a decrease in corpora lutea, an increase in the number of cystic follicles, and a thinning of follicular cells, which suggests that postnatal paraben exposure could adversely influence ovarian follicle development and thus potentially lead to diminished ovarian reserve. These changes could be a result of the estrogenic action of parabens. Similar effects have been observed in adult mice exposed to diethylstilbestrol (Hong et al. 2010).

In a study of pregnant rats treated subcutaneously with parabens, Taxvig et al. (2008) found a decrease in ER-β expression in the ovaries of female fetuses exposed to BP (MP and PP were not evaluated). In an *in vitro* study using MCF-7 human breast cancer cells, Okubo et al. (2001) found that ER-α expression decreased and progesterone receptor (PR) expression increased after administration of BP and isobutyl-paraben (MP and PP were not evaluated). It is possible that altered gene expression related to *in utero* paraben exposure could adversely affect the ovarian follicle pool. Proper estrogen signaling is a key component in the development of the ovarian follicle pool *in utero* (Crain et al. 2008), and disruption of this signaling could manifest as diminished ovarian reserve during a woman’s reproductive years.

In a previous study including the same patients from the MGH Fertility Center, we found that one urine sample was reasonably representative of urinary paraben concentrations over several months (intraclass correlation coefficients between 0.4 and 0.5 for MP, PP, and BP using nonpregnancy samples) (Smith et al. 2012). In the present study, because multiple samples were collected from some women, we used a summary exposure measure for each participant by taking the geometric mean of all urine samples collected prior to the outcome measure. Although one urine sample may reasonably represent several months of exposure, one strength of our study is that the collection of multiple samples should reduce exposure misclassification during that time period. However, a limitation of this study is that the time period of collection of the urine samples was up to 3 years before the outcome measure. It is unknown whether the window of exposure that is most etiologically relevant to the outcomes assessed is the year prior to the outcome measure, for example, or any earlier period in the life course (e.g., pubertal or even *in utero* exposure). If paraben exposure within several months prior to evaluation is the relevant window of exposure, the summary exposure measure used may reasonably represent the relevant exposure period. However, we believe that any exposure misclassification in this study is nondifferential. In future studies with larger sample sizes, we recommend examination of the time window to determine whether samples collected closer in time (i.e., a 3-month window) are more strongly associated with the outcome measures than are samples collected more remotely.

Another limitation of the present study is the relatively small sample size, which may limit our ability to detect an association. In addition, not all women had all three of the outcome measures because they are all not always clinically performed. However, this study is the first of its kind, and we suggest further investigation using a larger sample size to detect potentially subtle changes in markers of ovarian reserve in response to suspected endocrine-disrupting chemicals. Inclusion in our study of the high proportion of Caucasians and older women, as well as the sole inclusion of women from a fertility clinic undergoing *in vitro* fertilization or intrauterine insemination, all with varied SART diagnoses, may also limit the generalizability of these findings to non-Caucasians, younger women, and women with no difficulties conceiving. Because of the numerous xenoestrogens in personal care products, food, and medications (e.g. parabens, bisphenol A, benzophenone-3, triclosan), we suggest that future studies take into account the potential for the effect of estrogenic mixtures (e.g., assessing interactions between exposure categories of the chemicals). Finally, there is also the possibility of bias from uncontrolled confounding, given that personal care product use may change with age.

## Conclusion

The present study provides evidence suggesting that exposure to PP may lead to diminished ovarian reserve and contribute to ovarian aging among women at an infertility clinic. It has been estimated that in 2002 there were > 7 million women with impaired fecundity in the United States, and > 5 million women were reported as seeking help to become pregnant (Chandra et al. 2005). This is a large subpopulation of women that may be especially sensitive to endocrine-disrupting chemicals. Finally, although the parabens evaluated for the present study are considered to be safe (have a GRAS designation) based on a 1972 decision by the FDA (FDA 2013), given their widespread use and ubiquitous human exposure, further research using modern toxicologic designs and end points may be warranted. Our results suggest the need for future human studies to explore these associations in other populations with a larger sample size.

## Supplemental Material

(147 KB) PDF
