# Supplementary material for: Urinary Paraben Concentrations and Ovarian Aging among Women from a Fertility Center
Source: Environ Health Perspect. 2013 Aug 2;121(11-12):1299–305. doi: 10.1289/ehp.1205350 (PMC3855500; doi:10.1289/ehp.1205350)
Supplement: (147 KB) PDF [file ehp.1205350.s001.pdf]

Environmental Health Perspectives doi:10.1289/ehp.1205350

Supplemental Material

Urinary Paraben Concentrations and Ovarian Aging among Women from a Fertility Center

Kristen W. Smith, Irene Souter, Irene Dimitriadis, Shelley Ehrlich, Paige L. Williams, Antonia M. Calafat, and Russ Hauser

Table of contents:

Supplemental Material, Table S1. Estimated mean percent change in AFC by summed urinary paraben concentration tertile based on Poisson regression models .…………..……...…….Page 2

Supplemental Material, Table S2. Estimated mean change in day 3 FSH (IU/L) by summed urinary paraben concentration tertile based on linear regression models …………...….….Page 3

Supplemental Material, Table S3. Estimated mean percent change in OV by summed urinary paraben concentration tertile based on linear regression models ..………..…..……...…….Page 4

Supplemental Material, Table S1. Estimated mean percent change in AFC by summed urinary paraben concentration tertile based on Poisson regression model[a]

Summed Paraben Concentration	N	Estimated Mean Percent Change in AFC (95% CI)	p-value
**EEQ(parabens)[b]**			
Tertile 3 (57.3-522)	47	-6.4 (-24.4, 15.8)	0.54
Tertile 2 (19.2-56.9)	48	1.1 (-17.3, 23.5)	0.91
Tertile 1 (0.50-19.0)	47	0 (Reference)	
p-value for trend		0.54	
**Sum(parabens)[c]**			
Tertile 3 (3.09-19.0 μMol/L)	47	-10.8 (-28.2, 10.7)	0.30
Tertile 2 (1.13-3.07 μMol/L)	48	-6.5 (-23.4, 14.1)	0.51
Tertile 1 (0.039-1.12 μMol/L)	47	0 (Reference)	
p-value for trend		0.30	

[a]All model results adjusted for age. Paraben concentrations are SG-corrected.

[b]EEQ is the estrogen equivalency factor approach (sum of molar concentation of paraben times estrogen potency factor).

[c]Molar sum of parabens.

Supplemental Material, Table S2. Estimated mean change in day 3 FSH (IU/L) by summed urinary paraben concentration tertile based on linear regression models[a]

**Summed Paraben Concentration**	**N**	**Estimated Mean Change in FSH (95% CI)[a]**	**p-value**
**EEQ(parabens)[b]**			
Tertile 3 (54.7-522)	37	0.73 (-0.70, 2.16)	0.31
Tertile 2 (22.9-54.0)	37	0.25 (-1.21, 1.70)	0.74
Tertile 1 (0.50-22.3)	36	0 (Reference)	
p-value for trend		0.31	
**Sum(parabens)[c]**			
Tertile 3 (3.25-19.0 µMol/L)	37	0.54 (-0.89, 1.97)	0.46
Tertile 2 (1.31-3.16 µMol/L)	37	0.84 (-0.58, 2.27)	0.24
Tertile 1 (0.075-1.28 µMol/L)	36	0 (Reference)	
p-value for trend		0.46	

[a]The parameters estimates can be interpreted as a IU/L change in day 3 FSH for each tertile in summed paraben urinary concentrations relative to the first tertile. All model results are adjusted for age. Paraben concentrations are SG-corrected.

[b]EEQ is the estrogen equivalency factor approach (sum of molar concentation of paraben times estrogen potency factor).

[c]Molar sum of parabens.

Supplemental Material, Table S3. Estimated mean percent change in OV by summed urinary paraben concentration tertile based on linear regression model[a]

Summed Paraben Concentration	N	Estimated Mean Percent Change in OV (95% CI)	p-value
**EEQ(parabens)[b]**			
Tertile 3 (56.0-338)	36	-13.1 (-32.9, 12.6)	0.28
Tertile 2 (19.2-55.0)	37	9.9 (-15.0, 42.3)	0.47
Tertile 1 (0.62-18.2)	36	0 (Reference)	
p-value for trend		0.28	
**Sum(parabens)[c]**			
Tertile 3 (2.82-19.0 μMol/L)	36	-2.4 (-24.6, 26.4)	0.85
Tertile 2 (0.93-2.74 μMol/L)	37	23.6 (-4.7, 60.4)	0.11
Tertile 1 (0.054-0.93 μMol/L)	36	0 (Reference)	
p-value for trend		0.83	

[a]All model results adjusted for age. Paraben concentrations are SG-corrected.

[b]EEQ is the estrogen equivalency factor approach (sum of molar concentation of paraben times estrogen potency factor).

[c]Molar sum of parabens.
